# Surface Lipids in Nematodes are Influenced by Development and Species-specific Adaptations

**DOI:** 10.1021/jacs.4c12519

**Published:** 2025-02-12

**Authors:** Anna M. Kotowska, Fumie Hiramatsu, Morgan R. Alexander, David J. Scurr, James W. Lightfoot, Veeren M. Chauhan

**Affiliations:** † Advanced Materials & Healthcare Technologies Division, School of Pharmacy, University of Nottingham, University Park, NG7 2RD Nottingham, U.K.; ‡ Max Planck Research Group Genetics of Behavior, Max Planck Institute for Neurobiology of Behavior−caesar, Ludwig-Erhard-Allee 2, 53175 Bonn, Germany

## Abstract

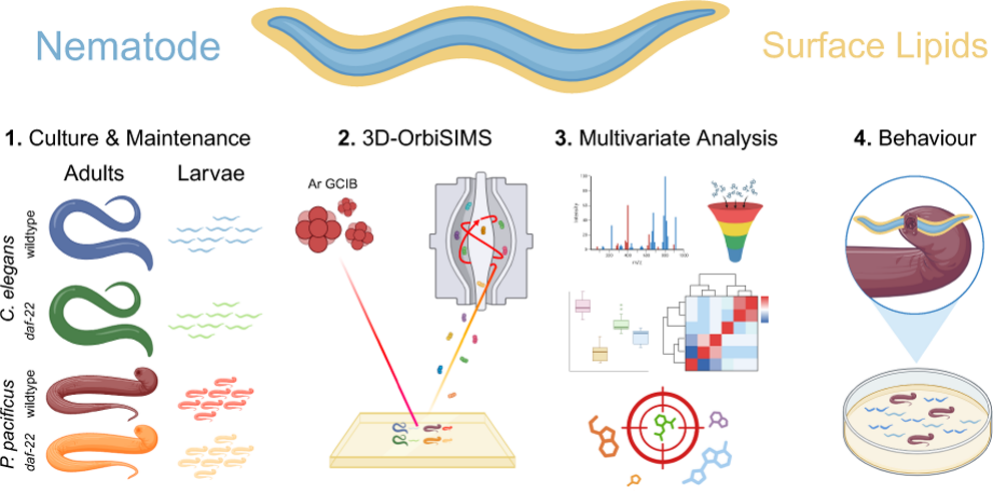

The surface of an organism is a dynamic interface that continually adapts to its environment. In nematodes, the cuticle forms a complex boundary that protects against the physicochemical pressures. However, the precise molecular composition and function of this surface remain largely unexplored. By utilizing 3D-OrbiSIMS, an advanced surface-sensitive mass spectrometry method, we directly characterized the molecular composition of the outermost regions (∼50 nm) of *Caenorhabditis elegans* and *Pristionchus pacificus* to improve the understanding of species-specific surface lipid composition and its potential roles in nematode biology. We found that nematode surfaces consist of a lipid-dominated landscape (>81% *C. elegans* and >69% *P. pacificus* of all surveyed chemistries) with distinct compositions, which enrich in granularity and complexity through development. The surface lipids are also species-specific, potentially highlighting distinct molecular compositions that are derived from diverging evolutionary paths. By exploring the effect of mutations on lipid production, we found the peroxisomal fatty acid β-oxidation component *daf-22* is essential for defining the surface molecular fingerprint. This pathway is conserved across species in producing distinct chemical profiles, indicating its fundamental role in lipid metabolism and maintaining the surface integrity and function. Furthermore, we discovered that variations in surface lipids of *C. elegansdaf-22* larvae contribute to significantly increased susceptibility to predation by *P. pacificus*. Therefore, our findings reveal that nematode surface lipids are developmentally dependent, species-specific, and fundamental in interspecies interactions. These insights pave the way for further exploration into the physiological and behavioral significance of surface lipids.

## Introduction

Organisms interact with their environment through surface components that play important roles in survival.^1^ These surfaces serve as dynamic interfaces, adapting continuously to physiological changes and environmental stimuli.^2^ However, due to the complex blend of different chemistries, deciphering the contribution of specific components can be challenging.

The nematode *Caenorhabditis elegans*, with its advanced genetic and molecular tools, serves as an important model organism for studying surface composition and its biological functions.^3,4^ This has enabled the elucidation of molecular mechanisms involved in the synthesis of various surface components and their biological activities. For example, specific surface proteins and glycoproteins have been identified, playing crucial roles in structural integrity and environmental responses.^5,6^ These components regulate developmental processes, such as molting and growth,^7,8^ and behaviors including locomotion^9^ and mating.^10^ Therefore, the nematode surface plays a complex, multifaceted role across diverse processes, and it is likely that the known components represent only a subset of its adaptation strategies.

The nematode cuticle and its surface coat represent the outermost layer, connecting the organism to its external environment. In free-living nematodes, the cuticle acts as a permeability barrier^11^ and must protect against both abiotic hazards such as desiccation,^12^ and biotic factors including pathogenic bacteria,^13^ fungal traps,^14^ and predatory nematodes.^15^ The cuticle comprises cross-linked collagens,^16^ glycoproteins, cuticulins, and lipids,^17^ which are synthesized by the hypodermal cells.^18^ Furthermore, as the nematodes undergo larval molts, their surface coats are replenished and replaced. The expression of many of these components is tightly regulated and oscillates in synchronicity with the organism’s development and molt cycle.^19^ Therefore, this sophisticated surface architecture underpins not only the mechanical properties of these organisms but also their chemical landscape, influencing interactions critical for survival.

Despite the identification of several surface proteins,^20−22^ the exact chemical composition and the broader significance of the nematode surface in supporting physiology and behavior remain largely uncharted. This knowledge gap arises not from an absence of curiosity but from the limitations in available tools for accurately capturing, analyzing, and interpreting molecular surface chemistry.

## Results

### Development Influences Surface Lipids

**Figure 1:**
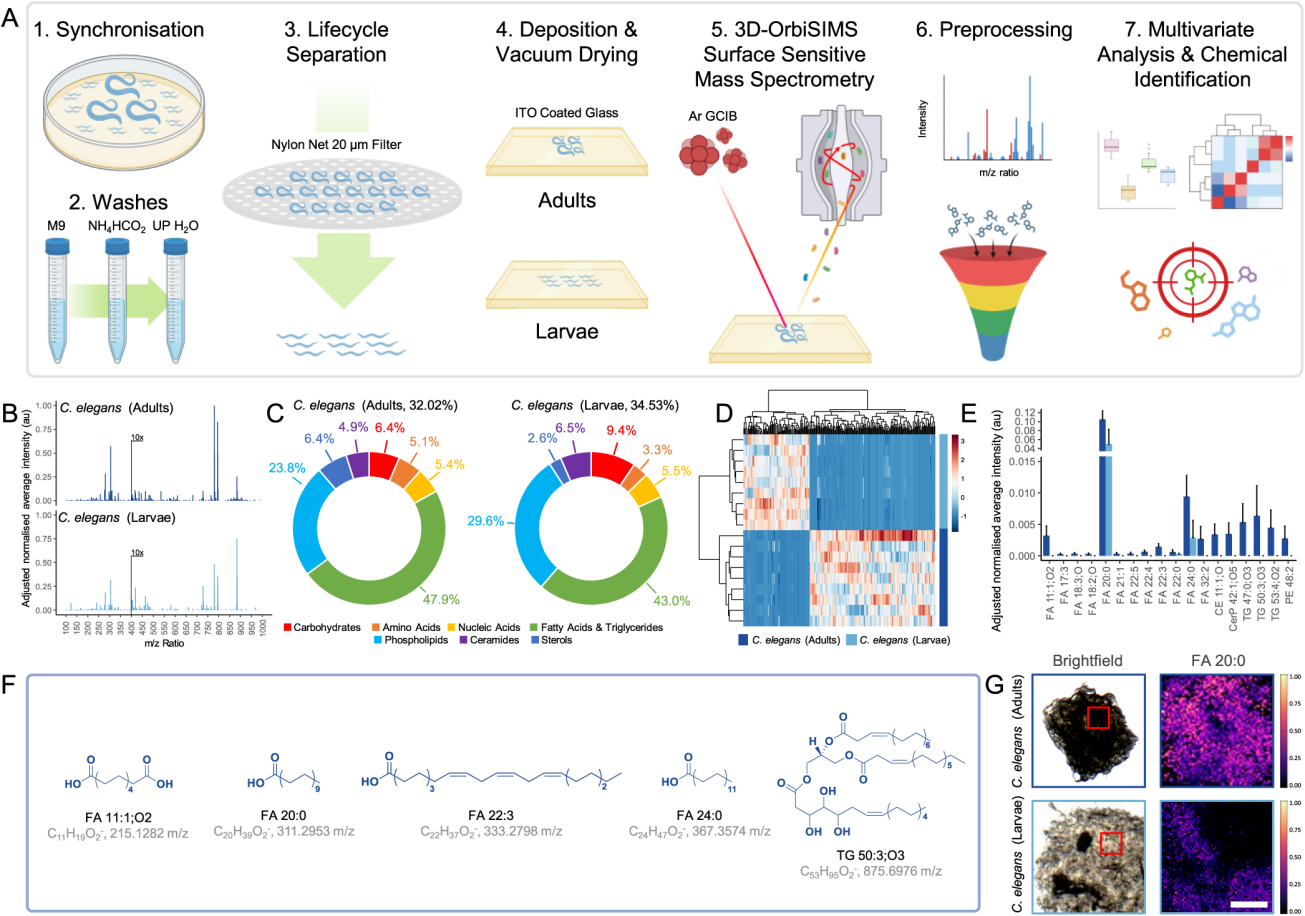
Surface-specific chemistry for *C. elegans* adults and larvae evaluated using 3D-OrbiSIMS. (A) Schematic detailing capture of adults or larvae’s surface chemical maps using 3D-OrbiSIMS (100 μm^2^, *n* = 9, created with BioRender.com). (B) Averaged *C. elegans* adults and larvae surface secondary ion mass spectra, normalized to maximum intensity across spectra, where intensity *m*/*z* > 400 enhanced 10× for visibility. (C) Distribution of molecular assignments determined using chemical filtration (Table S1), as a percentage of total ions surveyed. (D) Hierarchical clustering heatmap of *m*/*z* ratios for *C. elegans* adults and larvae (rows, *n* = 9), based on PC1 loadings greater than one standard deviation from the mean, showing clustering of similar surface chemistries with scaled normalized intensity (columns). (E) Significantly different chemistries on *C. elegans* adults and larvae’s surfaces (*P* < 0.001 by Student’s *t*-test, *n* = 9) present in LIPIDS MAPS with putative (F) chemical assignments h and structures. (G) Representative normalized intensity maps of *C. elegans* adult and larva surface chemistry, scale = 100 μm.

The study of nematode surfaces has traditionally relied upon methods such as liquid chromatography–mass spectrometry (LC–MS) for analyzing homogenates and surface extractions.^23^ Time-of-flight (TOF) based mass spectrometry techniques, like matrix-assisted laser desorption/ionization (MALDI)^24^ and secondary ion mass spectrometry (SIMS),^25,26^ have also been used to directly analyze surfaces, although these provide relatively lower mass accuracy. Advancements in surface-sensitive mass spectrometry, such as the 3D-OrbiSIMS, which combines a gas cluster ion beam (GCIB, Ar_3000_^+^) with an Orbitrap analyzer,^27^ provide a significant uplift in the ability to understand the chemical complexity of biological samples,^28,29^ enabling direct surface chemical mapping with relatively high spatial resolution (≥2 μm) and mass resolving power (>240,000 at *m*/*z* 200), achieved in the absence of chemical fixation or additional labeling. Additionally, its field of view (500 μm × 500 μm) facilitates the imaging of the entire length of the nematode, enabling a comprehensive analysis of the organism’s chemical composition (Figure 1A). Using this technique, the focus was to investigate the composition of the *C. elegans* surfaces, which consists of rich topological features that generate the organism’s external morphology (Figure S1). Through control of the ion dose (2.70 × 10^14^ ions/cm^2^), the surface analysis was confined to the outermost regions (approximately 50 nm in depth^30^), corresponding to the cortical cuticle of *C. elegans*.^31^ Global analysis of adult and newly hatched L1 larvae mass spectra suggested that their surfaces are very similar (Figures 1B and S2). Distribution of molecular assignments into chemical classes (Figure 1C and Table S1) revealed the OrbiSIMS spectra for both the adult and larvae *C. elegans* cuticle coats were dominated by lipids tightly bound to the cuticle surface, referred to as surface-anchored. Specifically, the total lipid composition, which includes fatty acids and triglycerides, phospholipids, ceramides, and sterols, contributed to 83.11% and 81.75% of the OrbiSIMS spectra for adults and larvae, respectively. Principal Component Analysis (PCA, Figure S3) and hierarchical clustering heatmaps (Figure 1D) delineated the developmental stages of *C. elegans* and also initiated the categorization of distinct chemical profiles responsible for the observed differences in surface chemistry.

Secondary ions exhibiting significant intensity differences across developmental stages (*P* < 0.001, Student’s *t*-test, *n* = 9) were evaluated using the LIPID Metabolites And Pathways Strategy (LIPID MAPS) database^32^ (Figure 1E). Furthermore, based on exact mass data aligning with Level 3 confidence, which permits tentative structure identification through contextual evidence,^33^ we proposed putative lipid structures to aid in interpreting surface chemistries (Figure 1F), relying on the most likely isobaric assignments derived from LIPID MAPS (Supporting Information Spreadsheet S1). These lipid double bond configurations are likely in *cis*-orientations, as nematode desaturase enzymes are known to introduce *cis* double bonds during lipid biosynthesis.^34−36^ Overall, there was a notable increase in the complexity of surface chemistries in adults, characterized by the prevalence of longer-chain fatty acids and triglycerides, which were seldom detectable in larvae. While certain fatty acids, such as FA 11:1;O2, 20:0, 22:0, and 24:0 (Figure 1F), were identified in both developmental stages, their intensities remained significantly higher on the surface of adults. Representative normalized intensity maps visually highlight the differences in surface chemistry between *C. elegans* adults and larvae, with FA 20:0 displaying the most pronounced relative intensity across the two developmental stages (Figure 1G). These observations, obtained using 3D-OrbiSIMS, provide important mechanistic insights into the dynamic and regulated nature of surface composition, highlighting the critical role of surface lipids in developmental transitions, which may also be important for developmental stage-specific physiology and behavior.

### Exploring Evolutionary Adaptations

**Figure 2:**
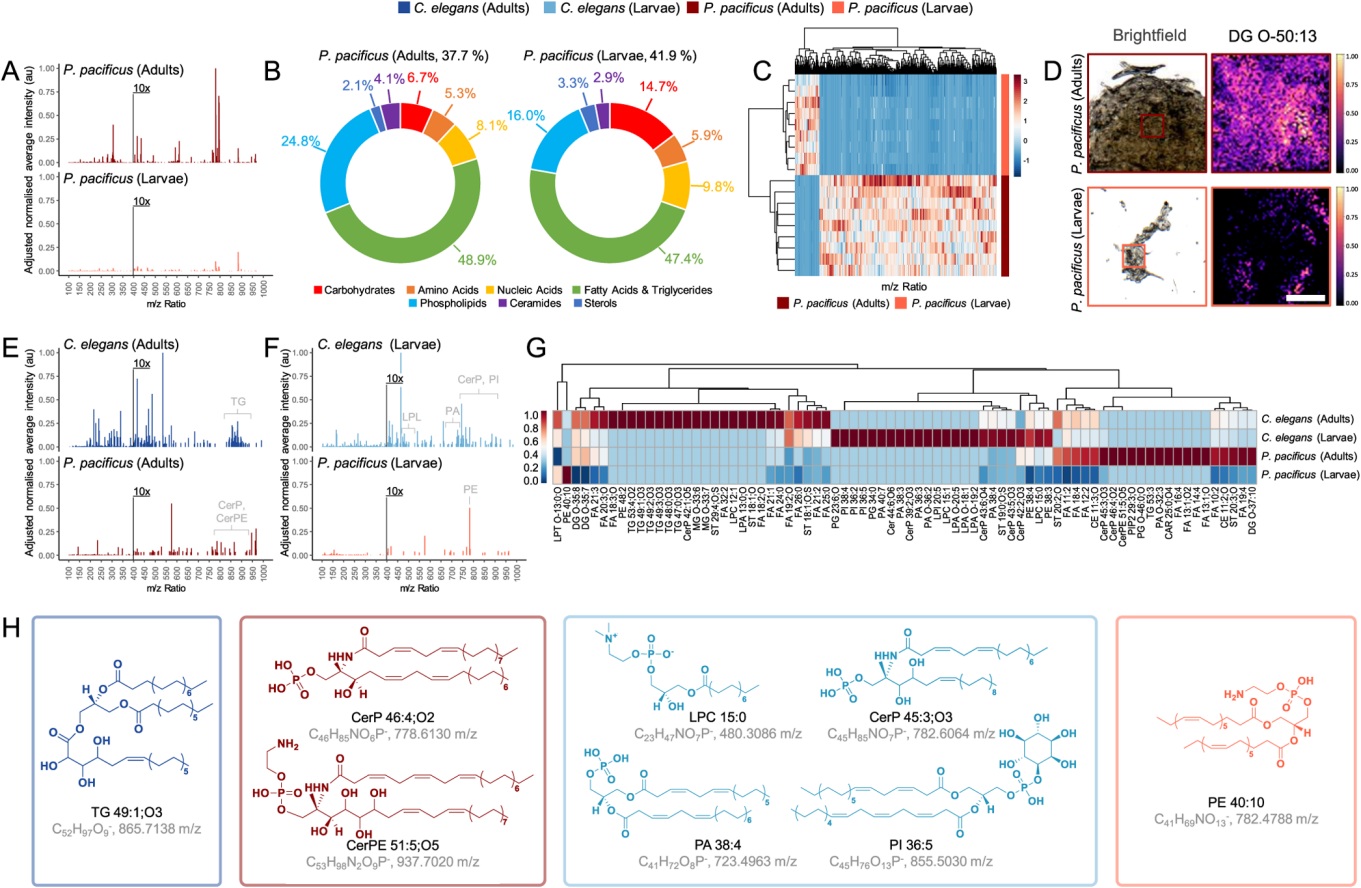
Surface chemistries are developmental stage dependent. (A) Averaged *P. pacificus* adults and larvae surface secondary ion mass spectra, normalized to maximum intensity across spectra, where intensity *m*/*z* > 400 enhanced 10× for visibility. (B) Distribution of molecular assignments determined using chemical filtration (Table S1), as a percentage of total ions surveyed. (C) Hierarchical clustering heatmap of *m*/*z* ratios for *P. pacificus* adults and larvae (rows, *n* = 9), based on PC1 loadings greater than one standard deviation from the mean, showing surface chemistry similarities with scaled normalized intensity and clustering of similar chemistries (columns). (D) Representative normalized intensity maps of *P. pacificus* adults and larvae surface chemistry, scale = 100 μm. Averaged surface secondary ion mass spectra exclusive to *C. elegans* and *P. pacificus* (E adults and F larvae). (G) Hierarchical clustering heatmaps of the distribution of exclusive chemistries for *C. elegans* (adults and larvae) and *P. pacificus* (adults and larvae), where clustering indicates potential shared regulation of exclusive chemicals and their relative intensity on nematode surfaces. (H) Putative structures of significantly different chemistries on *C. elegans* and *P. pacificus* surfaces (*P* < 0.001 by Student’s *t*-test, *n* = 9), present in LIPIDS MAPS.

While *C. elegans* is predominantly found in rotting fruit and is the most well-studied nematode species,^37^ the phylum Nematoda boasts a wide diversity and encompasses species that have adapted to a variety of ecological niches. For instance, *Pristionchus pacificus*, a nematode distantly related to *C. elegans*, is frequently associated with scarab beetles.^38^ Importantly, the surface properties of nematodes, being in direct contact with their environment, are likely to reflect evolutionary adaptations that are essential for thriving in specific habitats. For example, *P. pacificus* displays a unique topological surface arrangement when compared to *C. elegans* (Figure S1). Therefore, we conducted direct surface analysis of *P. pacificus* using 3D-OrbiSIMS to explore potential species-specific chemical signatures (Figures 2A, S4). The OrbiSIMS spectra for both *P. pacificus* adults and larvae were again dominated by surface-anchored lipids (Figure 2B). Collectively, the total lipid components contributed to 79.96% and 69.61% of the OrbiSIMS spectra for adults and larvae, respectively. Both PCA (Figure S5) and hierarchical clustering (Figure 2C) effectively differentiated the developmental stages of *P. pacificus*, revealing a reduction in the number of distinct mass ions characterizing *P. pacificus* larvae compared to those observed in *C. elegans*. Molecular assignments and putative chemical structures reveal *P. pacificus* adults possess significantly more complex surface chemistries than larvae (Figure S5). The predominant constituents are diglycerides (*e.g.,* DG O-50:13), sterols (*e.g.,* ST 20:0;O2), and a mix of unsaturated (*e.g.,* FA 12:2) and saturated fatty acids (FA 20:0, FA 22:0, and FA 24:0). Representative normalized intensity maps of DG O-50:13, showcase the most pronounced relative intensity differences (Figure 2D). This emphasizes the observed variations in surface chemistry between *P. pacificus* adults and larvae and further delineates the developmental distinctions. Therefore, these observations reveal that stage-dependent chemical compositions are also evident in other evolutionarily diverse nematode species.

### Species-specific Surface Chemistries

Having successfully identified the surface chemistries across developmental stages in two evolutionarily divergent nematodes, we next investigated species-specific differences in the surface composition. The distribution of molecular assignments revealed significant variations in lipid composition between *C. elegans* and *P. pacificus* (Figures 1C and 2B, respectively). In particular, the total lipid composition in *C. elegans* was consistently higher, with adults and larvae showing 3.75% and 14.84% greater lipid assignments, respectively, compared to *P. pacificus*. Specifically, the phospholipid composition in *C. elegans* larvae was 13.63% greater than that in *P. pacificus* larvae, indicating species-specific and developmental stage lipid adaptations. These differences were further evidenced by PCA (Figure S6) and hierarchical clustering heatmaps (Figure S7), which confirmed species-specific and developmentally distinct surface chemistries. Therefore, this suggests that *C. elegans* maintains higher lipid levels across developmental stages, unlike *P. pacificus*, where the lipid content was considerably reduced in larvae.

By studying lipid components exclusive to each developmental stage and species, we discovered a unique cluster of secondary ions in *C. elegans* adults, ranging from *m*/*z* 850–900 (Figure 2E), putatively identified as triglycerides (*e.g.,* TG 48:0;O3, TG 49:2;O3, and TG 49:1;O3, Figure S8). Whereas *P. pacificus* adults present secondary ions identified as ceramide phosphates (*e.g.,* CerP 46:4;O2) and phosphoethanolamine (e.g., CerPE 51:5;O5) in the higher mass ranges. *C. elegans* larvae surfaces, compared to *P. pacificus* larvae (Figure 2F,G,H), were also rich in ceramide phosphates (e.g., CerP 43:5,O3, CerP 43:6;O4, and CerP 45:3;O3) as well as a mixture of phosphatidylinositol (*e.g.,* PI 36:5), phosphatidic acids (*e.g.,* PA 38:4), and lysophosphatidic and lysophosphatidylcholine (Figures 2G,H, S8). *P. pacificus* larvae in general were absent of surface-specific lipids, compared to *C. elegans* larvae, except for a secondary ion at *m*/*z* 782.4788, which was putatively identified as phosphatidylethanolamine (PE 40:10, Figures 2G,H, S8). The observed reduction in lipid content on *P. pacificus* larvae compared to *C. elegans* larvae, and the absence of significant upregulation of surface-specific lipids, could be attributed to the unique developmental stage at which *P. pacificus* larvae hatch (J2 stage). This could contribute to a relatively naïve lipid surface, lacking the complex lipid profiles typically observed in later developmental stages and related species. Therefore, while the nematodes share common surface components and biochemical pathways, these analyses highlight species-specific adaptations across divergent evolutionary paths, enhancing our understanding of ecological diversity.

### Surface Chemistries being *daf-22*-Dependent

**Figure 3:**
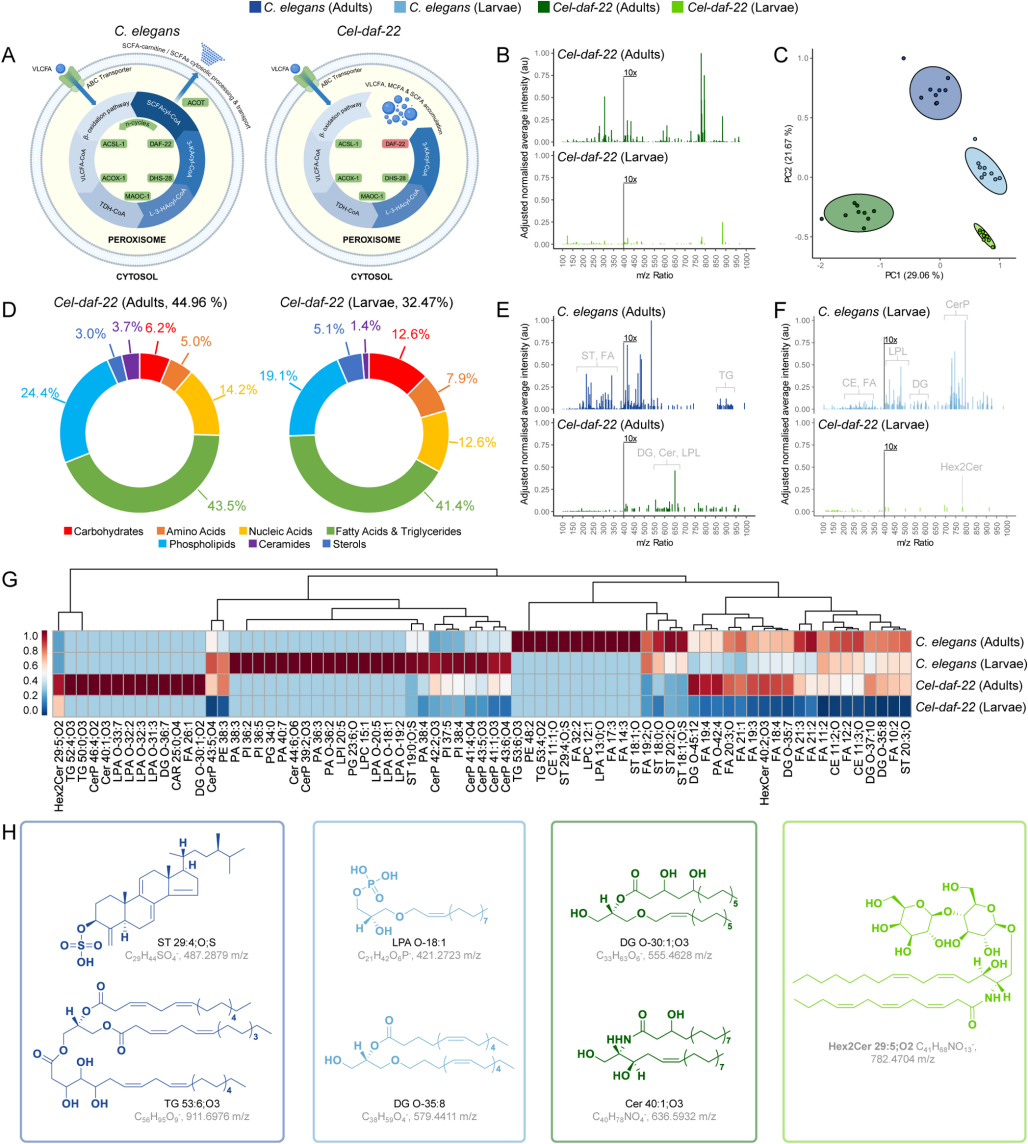
*C. elegans* surface profile is dependent on *daf-22*. (A) Schematic of peroxisomal β-oxidation pathway in wildtype and *daf-22* mutation, restricting VLCFA, MCFA, and SCFA processing and transport (created with BioRender.com). (B) Averaged *Cel-daf-22* adults and larvae surface secondary ion mass spectra, normalized to maximum intensity across spectra, where intensity *m*/*z* > 400 enhanced 10× for visibility. (C) PCA PC1 and PC2 scores plot for *C. elegans* wildtype and *Cel-daf-22* developmental nematode stages. (D) Distribution of molecular assignments determined using chemical filtration (Table S1), as a percentage of total ions surveyed. Averaged surface secondary ion mass spectra exclusive to *C. elegans* and *Cel-daf-22* [(E) adults and (F) larvae]. (G) Hierarchical clustering heatmaps of the distribution of exclusive chemistries for *C. elegans* (adults and larvae) and *Cel-daf-22* (adults and larvae), where clustering indicates potential shared regulation of exclusive chemicals and their relative intensity on nematode surfaces. (H) Putative structures of significantly different chemistries on *C. elegans* wildtype and *daf-22* mutant surfaces (*P* < 0.001 by Student’s *t*-test, *n* = 9), present in LIPIDS MAPS. *Acronyms:* ABC: ATP-binding cassette, ACOT: Acyl-CoA thioesterase, ACOX-1: Acyl-CoA oxidase 1, ACSL-1: Acyl-CoA synthetase long-chain family member 1, DAF-22:3-ketoacyl-CoA thiolase, DHS-28:3-hydroxyacyl-CoA dehydratase, L-3-HA-CoA: L-3-Hydroxyacyl-CoA, MAOC-1: Enoyl-CoA hydratase, CFA: Medium-chain fatty acids, SCFA: Short-chain fatty acids, TDH-CoA: Thiolase-CoA, VLCFA: Very long-chain fatty acids, and 3-KA-CoA: 3-Ketoacyl-CoA.

As surface-anchored lipids were prominent on the *C. elegans* cuticle surface, we explored the role of metabolic pathways in producing these chemistries by analyzing mutants in the peroxisomal β-oxidation pathway. This pathway is essential for the degradation of very long-chain fatty acids and the synthesis of short-chain fatty acids and ascarosides,^39−41^ with the thiolase DAF-22 acting as the terminal enzyme (Figure 3A).^42,43^ Therefore, we examined *daf-22* mutants in *C. elegans* to assess their impact on surface chemistries across both adult and larval stages (Figure 3B). 3D-OrbiSIMS analysis revealed that *daf-22* mutants lacked many surface chemistries present in both wildtype adults and larvae. PCA effectively differentiated between the wildtype and *daf-22* mutants, which is consistent with substantial alterations to the surface chemistries in *daf-22* animals (Figures 3C and S9). Analysis of the distribution of molecular assignments highlighted that *Cel-daf-22* adults and larvae exhibited an abundance of surface-anchored lipids, akin to wildtype strains (Figure 3D). The total lipid component for *Cel-daf-22* adults and larvae was reduced by 8.5% and 14.85% in adults and larvae, respectively, as in the percentage of lipid components compared to *C. elegans* wildtypes. In order to identify the precise effects of the *daf-22* mutations on the *C. elegans* surface composition, chemistries were isolated that were exclusive to either adult *C. elegans* wildtype or *daf-22* mutants (Figure 3E,F). Adult *Cel-daf-22* mutants exhibited an absence of higher mass complex surface chemistries (*m*/*z* 850–950), putatively identified in wildtypes as unsaturated triglycerides (*e.g.,* TG 53:4;O2 and TG 53:6;O3, Figure S10), as well as a reduction in the relative abundance of lower mass ions *m*/*z* < 400 consisting of unsaturated sterols and fatty acids (Figure 3E). Instead, an accumulation of mass ions between *m*/*z* 550–650 was found, putatively identified as diglycerides (*e.g.,* DG O-30:1;O2) and ceramides (*e.g.,* Cer 40:1;O3), as well as unsaturated lysophosphopholipids (Figure 3G,H). The larval OrbiSIMS surface composition of *Cel-daf-22* mutants also differed in a substantial number of chemistries when compared to wildtype larvae at all *m*/*z* ratios (Figure 3F,G). In particular, there was an absence of ceramidephosphates between *m*/*z* 700 and 800, lysophosphopholipids between *m*/*z* 400 and 500, and cholesteryl ester and unsaturated fatty acids between *m*/*z* 200 and 350 (Figures 3G,H, S10). The diglycosylceramide Hex2Cer 29:5;O2 was also putatively found to have higher relative intensity in *Cel-daf-22* adults and larvae (Figure 3G,H). Complex glycosphingolipids have been shown to modulate signal transduction pathways and influence behavior, and simpler diglycosylceramides may have a similar function.^44^ Therefore, the peroxisomal β-oxidation pathway is required for the synthesis of surface-anchored lipids and the establishment of stage-specific surface compositions in *C. elegans* development.

Given the divergent chemistries observed on *C. elegans* and *P. pacificus* surfaces, we also investigated the importance and conservation of the peroxisomal β-oxidation pathway for establishing the surface composition of *P. pacificus* using 3D-OrbiSIMS. Specifically, *P. pacificus* possesses two *daf-22* homologues, *Ppa-daf-22.1* and *Ppa-daf-22.2*.^45^ Analysis of the distribution of molecular components highlighted negligible differences in total lipid composition (<1%) for both *Ppa-daf-22.1/2* double mutant adults and larvae, compared to wildtypes (Figure S11). However, *Ppa-daf-22.1/2* mutants exhibited a reduction in specific secondary ions and their relative intensity (Figures S12 and S13), indicating that although the overall composition remains largely unchanged, certain lipids are affected. This contrasts with the findings observed on *C. elegans* surfaces, where substantial changes in overall lipid composition were identified in *daf-22* mutants. Our observations suggest that *P. pacificus* may have compensatory mechanisms that maintain overall surface lipid levels despite disruptions in the peroxisomal β-oxidation pathway. Therefore, these findings highlight differences in the regulatory mechanisms of surface lipid composition between these species and provide insight into the evolutionary adaptations of the lipid metabolism pathways in nematodes.

### Surface Chemistries Regulate Behaviors

**Figure 4:**
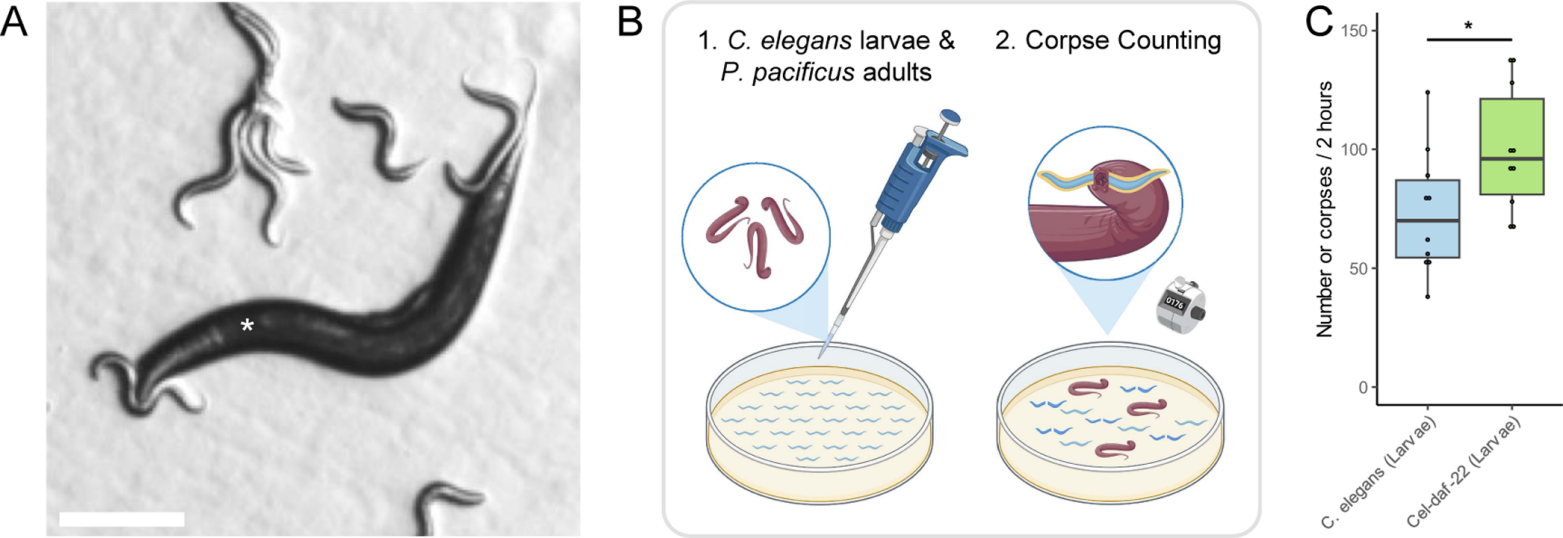
Surface chemistries regulating contact-dependent predatory behavior. (A) Representative images of *P. pacificus* wildtype (*) contact-dependent predatory biting behavior toward *C. elegans* wildtype larvae (scale = 100 μm). (B) Schematic of corpse assay of *P. pacificus* preying on *C. elegans* larvae (created with BioRender.com). (C) Predatory behavior of *P. pacificus* wildtype (adult) toward *Cel-daf-22* larvae (*P < 0.05—Student’s *t*-test, *n* = 10).

Finally, given the distinct surface chemical compositions observed between *C. elegans* and *P. pacificus*, we hypothesized that these surface chemistries may influence species-specific contact-dependent interactions. *P. pacificus* uses its phenotypically plastic teeth-like denticles to direct predatory behavior toward other nematode species (Figure 4A, Supporting Information Video S1) as well as other *P. pacificus* con-specifics, resulting in highly cannibalistic interactions.^15,46−50^ Crucially, there appears to be little influence from any secreted molecules on this behavior, which is instead determined by nose contact of the predator with the cuticle surface of a potential prey.^51^ Therefore, to study whether the altered surface lipid chemistries in *Cel-daf-22* influence these predatory interactions, we conducted well-established predation assays using *P. pacificus* adults (Figure 4B).^15,48^

A significant difference in predation was observed between *C. elegans* wildtype and *Cel-daf-22* larvae, with *P. pacificus* adults preying more on *Cel-daf-22 larvae* (*P* = 0.037, Student’s *t*-test, *n* = 10, Figure 4C). The increased predation of *Cel-daf-22* larvae likely stems from altered surface lipids and an apparent reduction in key lipids, as identified using OrbiSIMS analysis (Figure 3G). This suggests a potential protective contact-dependent function of these lipids. The reduction in key structural lipids, such as fatty acids, sterols, and glycosphingolipids, may compromise the integrity of the cuticle, rendering the mutants more vulnerable to physical attack, or alternatively, *daf-22* mutants may be more quickly identified as potential prey. Furthermore, the alteration in surface composition, which was shown to produce novel lipid groupings on mutants, could also disrupt membrane-dependent pathways, potentially resulting in an increased susceptibility to predation. Therefore, these findings confirm the multifaceted role of surface lipids in nematode survival, including their potential role in mediating interactions and defense mechanisms.

## Discussion

In this study, we have performed direct chemical analysis of the outermost 50 nm of the nematode surface using 3D-OrbiSIMS, and we have generated an in-depth profile across two developmental stages and two evolutionarily distinct species. This represents a significant advance on previous approaches, which utilized homogenate production, surface extraction steps,^23^ or relied on lower-resolution time-of-flight measurements.^25^ We reveal that the nematode surface profile is not a static entity but instead comprises a complex, lipid-dominated landscape, which is dynamically modified during the organism’s development. Moreover, our observation that specific surface lipids vary in abundance and composition across developmental stages and between species suggests that these lipids may have roles beyond structural components, potentially contributing to developmental processes and species-specific adaptations. The *C. elegans* adult surface is characterized by the prevalence of complex lipid molecules, including longer-chain fatty acids and triglycerides, which are much less common in the larvae. These changes correlate with the maturing nematode metabolism,^52^ resulting in developmental stage-specific chemical compositions that may be important for distinct population or environmental interactions. Furthermore, by comparing the surface composition across two distinct lineages of free-living nematodes, we also found the surface chemistry is species-specific, indicating its importance as an evolutionary adaptive trait. While triglycerides dominate the surface of adult *C. elegans*, the surface of *P. pacificus* is instead comprised of ceramide phosphates, phosphatidylinositol, and phosphatidic acids. In addition, the larval surface between species is also strikingly different, with the *P. pacificus* larval surface featuring fewer lipids contributing to its more naïve profile. This observation highlights developmental and possibly evolutionary differences in lipid composition across species, suggesting distinct functional roles that encourage further exploration.

*P. pacificus* preys on nonkin species such as *C. elegans*, indicating species-specific recognition events during interspecies interactions. While the SELF-1 peptide has been shown to mediate kin recognition within *P. pacificus* populations,^48^ our observation of species-specific differences in surface lipid composition suggests that lipids may contribute to mechanisms involved in the predation of nonkin species. The increased susceptibility of *C. elegans daf-22* mutants to predation underscores the protective role of an intact lipid profile. The *daf-22* mutation disrupts the surface lipid composition, which may impair the physical barrier function of the cuticle, leading to increased susceptibility to predation by *P. pacificus*. Understanding the full extent of surface lipid functions, including potential roles in chemical communication, opens opportunities for further investigation. For example, in insects, surface lipids not only prevent desiccation but also mediate communication regarding sex, age, reproductive status, and kinship.^53^

Therefore, our studies not only highlight the complexity and dynamism of nematode surface chemistry but also highlight differences associated with their developmental stages and species-specific adaptations. The use of advanced surface-sensitive mass spectrometry technology will enhance our understanding of surface lipid profiles but also paves the way for exploring subsurface chemistries and other complex behavioral interactions. For example, mapping complete metabolic processes across organisms could improve our understanding of host-parasitic nematode interactions. This may contribute to the development of novel strategies to overcome parasitic infections, thereby advancing public health interventions. We anticipate that expanding these approaches to a broader range of nematode species and environmental conditions will further elucidate the evolutionary significance of surface chemistries and their role in physiology and behavioral interactions.

## Materials and Methods

### Nematode Culture, Sample Preparation, and Behavioral Assays

#### Nematode Culture

All nematodes used were maintained on standard NGM plates on a diet of *Escherichia coli* OP50.

#### Nematode Strains Utilized

The following strains, *C. elegans* and *P. pacificus*, were used in this study. The *C. elegans* strains included N2, the wildtype, and DR476, which has the *daf-22* (m130) mutation. For *P. pacificus*, the strains used were PS312, the wildtype, RS2770 with *daf-22.1* (tu489) and *daf-22.2* (tu504) mutations.

#### Nematode Sample Preparation

Nematodes were maintained on nematode growth medium (NGM) agar and *E. coli* (OP50) at 20 °C.^54−56^ Synchronized growth cycles were prepared by harvesting eggs from the gravid females. Samples for analysis were gathered postsynchronization and after larval hatching from NMG agar plates (3 × 10 cm plates) using M9 buffer (10 mL, stored at 4 °C). Suspended nematodes were washed sequentially using centrifugation M9 (10 mL, 1500 rpm, 1 min × 3), ammonium formate (150 mmol, 4 °C, 1500 rpm, 1 min × 3), and ultrapure water (18.2 MΩ, 1500 rpm, 1 min × 3) discarding the supernatant. These extensive washing steps are designed to remove any loosely associated or secreted lipids. The molecular components analyzed using 3D-OrbiSIMS are those that remain tightly bound to the nematode cuticle and are termed “surface-anchored”, highlighting their strong association with the nematode surface. After the final ultrapure deionized water wash, nematodes were resuspended in ultrapure water and passed through two Nylon-20 μm filters (total volume 50 mL) to separate the adults from the larvae. Nematodes were pelleted by centrifugation; the worms were resuspended in a minimal volume of water. They were then deposited on indium tin oxide (ITO) slides (70–100 Ω); excess water was dabbed off, and they were vacuum-dried. Lastly, they were refrigerated (−80 °C) until analysis.

#### Corpse Assay

Methods for the corpse assay have been adapted from the work of Lightfoot et al.^57^ Briefly, Prey nematode cultures of *C. elegans* were grown on standard NGM plates supplemented with an *E. coli* OP50 lawn until freshly starved, resulting in an abundance of young larvae. These plates were washed with M9 and passed through two 20 μm filters to isolate larvae, which were collected in an Eppendorf tube. 1.0 μL of *C. elegans* larval pellet was transferred onto a 6 cm NGM unseeded plate. Five *P. pacificus* adult nematodes were transferred to plates of prey (larvae of wildtype *C. elegans* and *Cel-daf-22*). Assay plates were meticulously examined after 2 h for dead *C. elegans* larvae. These emptied corpses can be distinctly recognized by their immobility coupled with evident morphological aberrations such as leaking internal organs or absent worm segments.

### Data Acquisition

#### Scanning Electron Microscopy

Nematode specimens were initially synchronized to ensure uniform developmental stages. Postsynchronization, adult worms were washed multiple times with M9 Buffer to remove any residual bacteria. The cleaned worms were fixed using 4% paraformaldehyde for 2 h at room temperature. Following fixation, specimens were subjected to a series of dehydration steps using an increasing concentration of ethanol solutions (30%, 50%, 70%, 90%, and 100%) for 10 min each. The dehydrated samples were critical point dried to preserve their natural morphology and prevent shrinkage. Once dried, specimens were mounted onto carbon coated aluminum stubs. To improve the conductivity and image quality, a thin layer of gold was sputter-coated onto the specimens. The coated nematode samples were then analyzed under the SEM, focusing on the surface structures and morphology.

#### 3D-OrbiSIMS Data Acquisition

Calibration of the Orbitrap analyzer was performed using silver clusters, according to the protocol described by Passarelli et al.^27^ The Bi_3_^+^ liquid metal ion gun and ThermoFisher Tune software were executed for calibration. An Ar_3000_^+^ primary gas cluster ion beam (GCIB, 20 keV, 2 μm diameter, duty cycle set to 27.78%, and target current was 24 pA) was used for sample sputtering and ejection of secondary ions. The Q Exactive images were acquired using a random raster mode (field of view 300 × 300 μm, pixel size 5 μm, cycle time 200 μs, and optimal analyzer target −69.5 V). Argon gas flooding was in operation to facilitate charge compensation regulating the pressure in the main sample chamber to 9.0 × 10^–7^ bar throughout the analysis. The images were collected in negative polarity (*m*/*z* 75–1125) with constant injection time (500 ms) total ion dose per measurement (2.70  ×  10^14^ ions/cm^2^) and mass-resolving power (240,000 at *m*/*z* 200). Given the sputtering rate for organic materials, an ion dose of 3.00 × 10^14^ ions/cm^2^ was estimated to have analyzed a sample depth of approximately 50 nm.^30^

### Data Extraction, Management, and Filtration

#### Data Extraction as Well as Noise Identification and Elimination

For each sample type (*n* = 13) and region of interest (100 μm^2^), data was normalized to total ion count in SurfaceLab (version 7). Data was extracted and filtered by maximizing data in each ROI using *C. elegans* wildtype adult as a reference. Through evaluation of the minimum intensity across the spectra, noise was determined as intensity counts less than 5 × 10^–6^ au, which identified ∼2500 secondary mass ions per ROI. The minimum noise (<5 × 10^–6^ au) and number of peaks (2500 secondary mass ions) criterion was translated to all additional data sets. Peak lists were combined to facilitate a data comparison using a mass accuracy of 5 ppm. To eliminate noise generation during merged peak list production, data were surveyed to identify and eliminate newly generated noise with average noise intensity less than 5 × 10^–6^. The master peak list was composed of 11 different samples (adults and larvae for *C. elegans* wildtype, *Cel-daf-22, P. pacificus*, *Ppa-daf-22.1/2* as well as *E. coli*, agar, and ITO glass) each with 9 replicates (*n* = 9, 100 μm^2^) and 9563 different peaks producing >1.1 million data points.

#### Mass Spectra Data Management and Visualization

The master peak list was searched to facilitate comparative analyses. Peak lists for comparative data sets were generated by combining data and removing data associated with *E. coli*, Agar and ITO, ensuring a <5 ppm mass accuracy, while applying an aggressive baseline threshold of 5 × 10^–6^ au for ions normalized to total ion count across each data set. These multivariate analyses were executed in R, utilizing R Studio. Data were visualized as averaged secondary ion mass spectra, derived from ROI (*n* = 9). The maximum filtered intensity was normalized and for data exceeding 400 *m*/*z*, a 10× multiplier was applied to facilitate data interpretation.

#### Distribution of Molecular Assignments

The distribution of molecular assignments was conducted using Secondary Ion Mass Spectrometry Molecular Formula Prediction (SIMSMFP)^58^ and search criterion defined as carbohydrates (C_1–7_H_2–12_O_1–6_) proteins and amino acids (C_1–8_H_2–17_O_1–3_N_1_S_0–1_), nucleic Acids (C_1–8_H_2–9_O_1–6_N_1–3_P_1_), fatty Acids and triglycerides (C_2–50_H_4–90_O_1–4_), Phospholipids (C_4–40_H_8–80_O_4–8_N_1_P_1–2_), and sterols (C_10–25_H_9–44_O_1_), with mass deviations <5 ppm. Instances where direct comparison of biological components between samples was required (*e.g., daf-22* mutant lipid component analysis) SIMSMFP was conducted to identify molecular components (*e.g.,* fatty acids, triglycerides, and phospholipids) which were then subtracted between organisms to <5 ppm mass accuracy to determine differentiating mass ions.

#### Data Analysis and Packages

A range of R packages were used, each serving a specific purpose. SurfaceLab (Version 7) was used for data acquisition and manipulation, while R Studio (2022.07.02 + 576) provided an integrated development environment for R, the programming language version 4.2.2. The “dendextend” package (version 1.17.1) aided in visualizing and comparing hierarchical clustering trees. Data frame tools were handled by dplyr (version 1.1.2), and factoextra (version 1.0.7) and factomineR (version 2.8) were used for visualization and extraction in multivariate analysis. The study also employed “forcats”s, “ggforce” (version 0.4.1) to accelerate “ggplot2” (version 3.4.3), and “ggrepel” (version 0.9.3) for positioning nonoverlapping labels in “ggplot2”. Additional packages included “ggsignif” (version 0.6.4), “ggtext” (version 0.1.2), and “gplots” (version 3.1.3) for plotting enhancements, “heatmaply” (version 1.4.2) for interactive cluster heat maps, and “lattice” (version 0.20–45) for trellis graphics. The “pheatmap” package (version 1.0.12) was used for heatmap production, and “plotly” (version 4.10.2) was used for interactive web graphics. The study also utilized “readr” (version 2.1.4) for reading rectangular data, “scales” (version 1.2.1) for scaling and formatting axis labels, “scatterplot3d” (version 0.3–44) for 3D scatter plots, “stringr” (version 1.5.0) for string operations, “svglite” (version 2.1.1) for SVG graphics, “tibble” (version 3.2.1) as a modern data.frame variant, and “tidyr” (version 1.3.0) for data tidying. The “tidyverse” collection (version 2.0.0) provided a comprehensive suite of data science packages, while “viridis” (version 0.6.3) and “viridisLite” (version 0.4.2) were used for creating perceptually uniform color maps.

## Statistical Analyses

### Statistical Analyses Performed in R Using Packages

#### Comparative Peak Analyses

Student’s *t*-test (two-tailed, unequal variance) was performed to identify significantly different mass during binary comparisons of data sets. Standard deviation was calculated between ROI (*n* = 9) and plotted as error on accompanying bar charts. Significantly different data were categorized based on their *p*-value thresholds such that * = *P* < 0.05, ** = *P* < 0.01, and *** = *P* < 0.001.

#### Principal Component Analyses

Principal Component Analysis (PCA) was conducted on comparative data sets, scaling, standardizing, and centering each variable so that they have a standard deviation of 1 and a mean of 0, respectively. This step was important to ensure that all variables contribute equally to the principal components, eliminating any undue influence from variables. For 2D and 3D scores, plots ellipses were used to visualize the 95% confidence interval to provide a guide to the consistency of data groupings.

#### Hierarchical Clustering Heat Map Analyses

Hierarchical clustering heatmap analysis utilized a data reduction approach by selecting only mass ions with principal component loadings that were greater than one standard deviation from the mean. This data reduction was key to data visualization, ensuring a focused analysis on the organisms and *m*/*z*-based separation of variables. Visualizations were optimized though the application of heatmaps for columns of data comparing strains.

#### Putative Structural Assignments of Surface Lipids

Chemistries exhibiting significant differences were probed by searching LIPIDMAPS with a mass deviation of <2.0 ppm (136 assignments, 90.7%) for the highest confidence in exact mass matching and 2.0–3.5 ppm (14 assignments, 9.3%) for high confidence matches with contextual insight. Proposed putative structures were based on exact mass data aligning with Level 3 confidence criteria,^33^ relying on the most likely isobaric assignments derived from LIPID MAPS. These lipids were assessed for likely *cis*-configurations, consistent with the enzymatic activity of nematode desaturases known to introduce *cis* double bonds during lipid biosynthesis.^34−36^ A comprehensive list of isobaric assignments and putative structures is provided in Supporting Information Sheet S1 to aid in interpreting surface chemistries.

## Data Availability

All the data are publicly available via the Nottingham Research Data Management Repository at DOI: 10.17639/nott.7386.

## Supporting Information Available

*C**. elegans* and *P. pacificus* rich topological features that generate the organism’s external morphology, *C. elegans* mass spectra optimization, multivariate analysis of *C. elegans* adults and larvae, *P. pacificus* mass spectra optimization, multivariate analysis of *P. pacificus* adults and larvae, multivariate analysis of *C. elegans* and *P. pacificus* developmental stages, comparing *C. elegans* and *P. pacificus* hierarchical clustering heatmaps, species-specific surface chemical contrast divergence, multivariate analysis of *C. elegans* wildtype and *daf-22* for each developmental stage, putative *C. elegans* wild-type and *daf-22* mutant chemical assignments and structures, *P. pacificus* surface profile is dependent on *daf-22*, *P. pacificus* surface profile is dependent on *daf-22*, multivariate analysis of *P. pacificus* wildtype and *daf-22* for each developmental stage, and elemental composition ranges of key biomolecules (PDF)
Adult *P. pacificus* wildtype predating on C. elegans wildtype larvae (MP4)
Isobaric assignments for nematode surface lipid composition (XLSX)
