# Supplementary material for: Surface Lipids in Nematodes are Influenced by Development and Species-specific Adaptations
Source: J Am Chem Soc. 2025 Feb 12;147(8):6439–49. doi: 10.1021/jacs.4c12519 (PMC11869268; doi:10.1021/jacs.4c12519)

Supporting Information

Surface lipids in nematodes are influenced by development and species-specific adaptations

Anna M. Kotowska [1], Fumie Hiramatsu [2], Morgan R. Alexander [1], David J. Scurr [1], James W. Lightfoot [2], Veeren M. Chauhan [1 *]

[1] Advanced Materials and Healthcare Technologies Division, School of Pharmacy, University of Nottingham, University Park Nottingham, NG7 2RD, UK

[2] Max Planck Research Group Genetics of Behavior, Max Planck Institute for Neurobiology of Behavior – caesar, Ludwig-Erhard-Allee 2, 53175, Bonn, Germany.

* Corresponding author Veeren M. Chauhan

Email: veeren.chauhan@nottingham.ac.uk (VMC)

This PDF file includes:

- Supplementary Figures 1 to 13
- Supplementary Table S1
- Supplementary Spreadsheet S1 Caption
- Supplementary Video S1 Caption

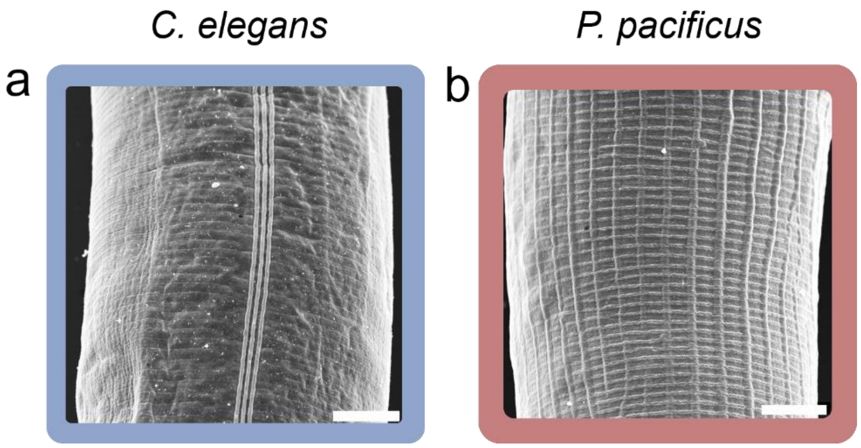

Fig. S1 | *C. elegans* and *P. pacificus* rich topological features that generate the organism's external morphology . SEM image of **a** C. elegans and **b** *P. pacificus* surface cuticle, scale = 8 µm.

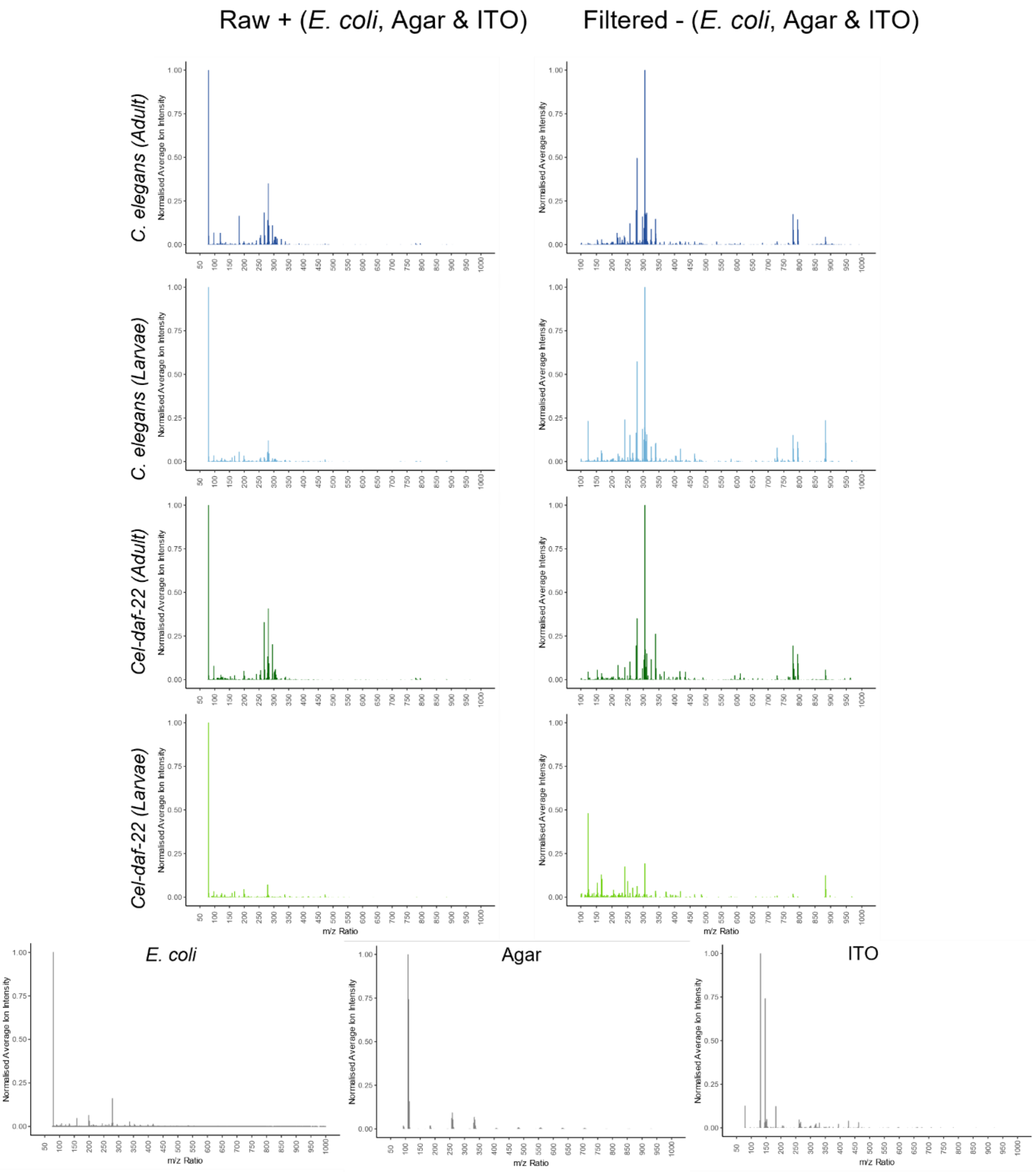

Fig. S2 | *C. elegans* mass spectra optimisation. Raw and filtered secondary ion mass spectra, eliminating *E.coli*, Agar and ITO, ions to 5 ppm.

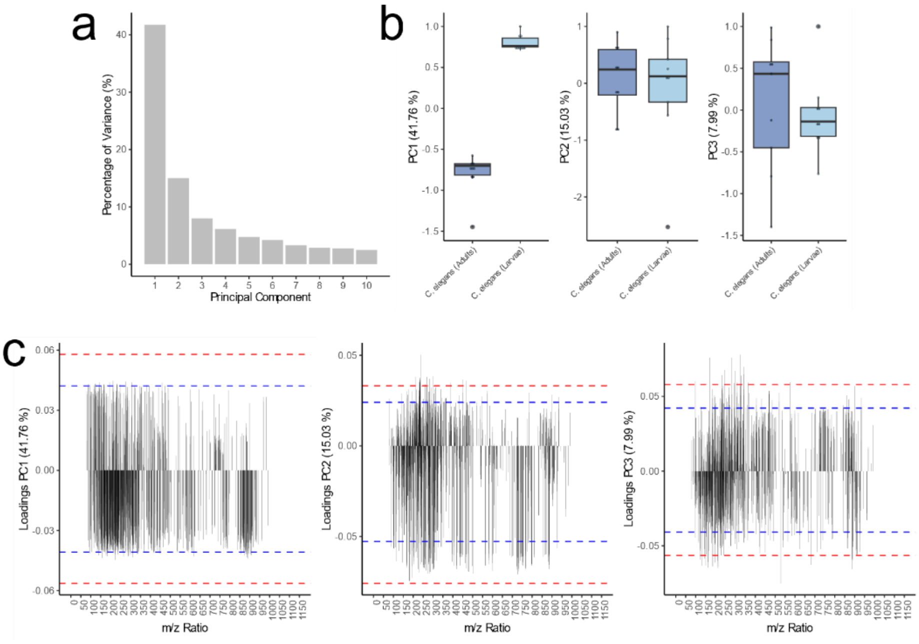

Fig. S3 | Multivariate analysis of *C. elegans* adults and larvae. a Percentage of variance on each principal component. **b** PC1, PC2 and PC3 b scores plot. **c** PC1, PC2 and PC3 loadings, where blue and red dashed lines indicate 1 and 2 standard deviations from the mean, respectively.

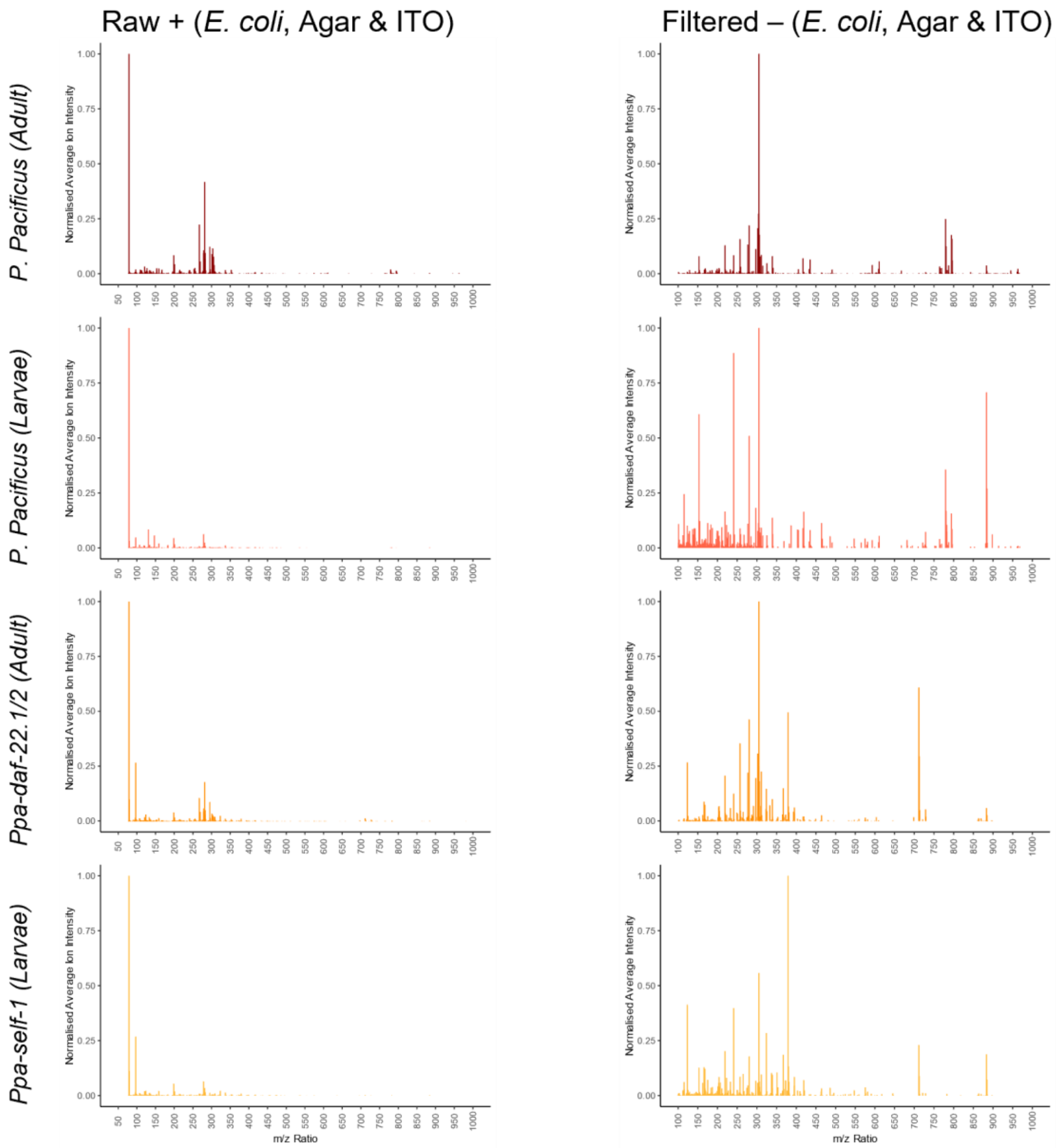

Fig. S4 | *P. pacificus* mass spectra optimisation. Raw and filtered secondary ion mass spectra, eliminating *E.coli*, Agar and ITO, ions to 5 ppm.

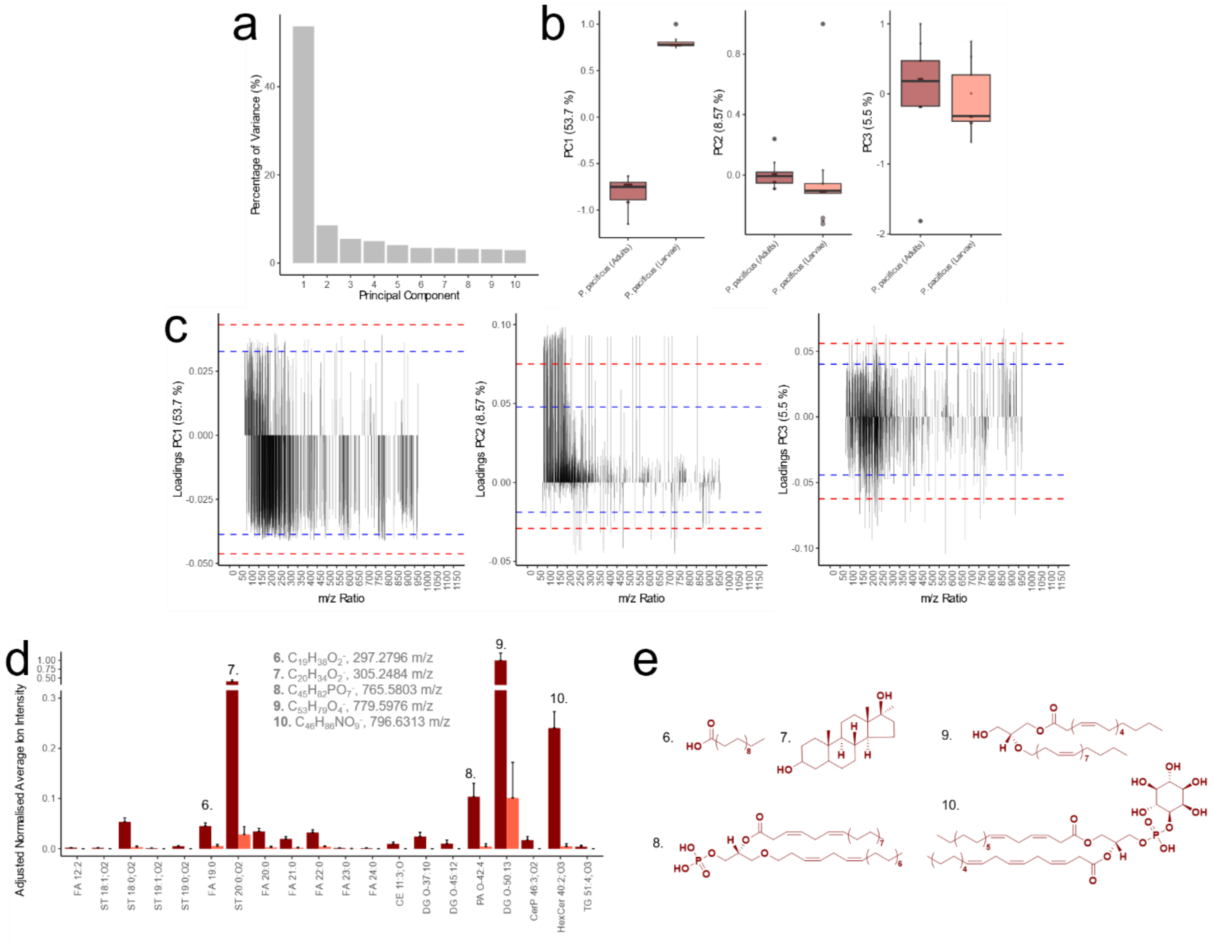

Fig. S5 | Multivariate analysis of *P. pacificus* adults and larvae. a Percentage of variance on each principal component. **b** PC2 and PC3 scores plot. **c** PC1, PC2 and PC3 loadings, where blue and red dashed lines indicate 1 and 2 standard deviations from the mean, respectively. Significantly different chemistries on *P. pacificus* adults and larvae surfaces, where (*P*<0.001 by Student's t-test, n=9), present in LIPIDS MAPS (M-H, < 2 ppm) with putative **d** chemical assignments and **e** structures.

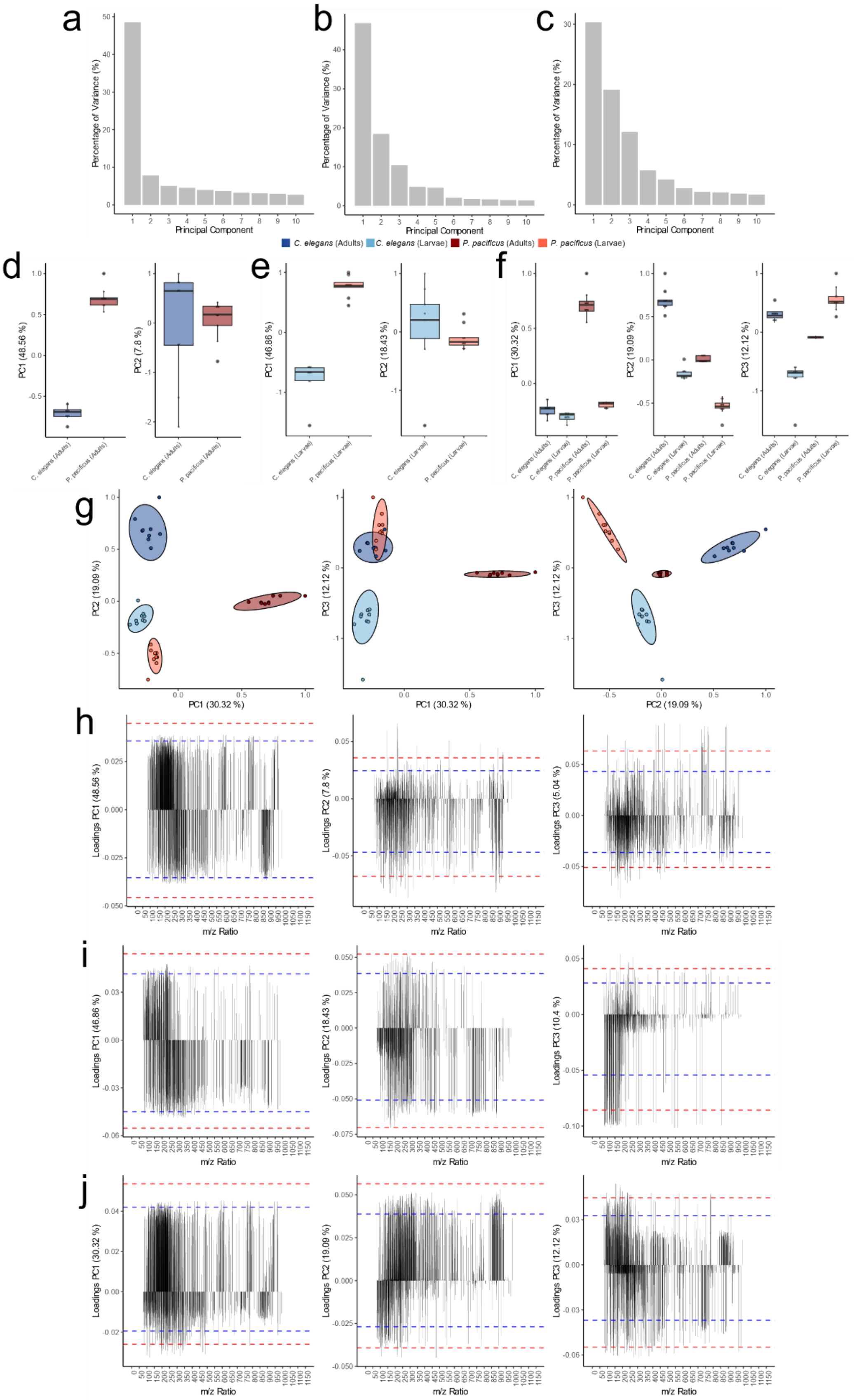

Fig. S6 | Multivariate analysis of *C. elegans* and *P. pacificus* developmental stages. a Percentage of variance on each principal component (**a** adults **b** larvae and **c** adults & larvae). PC1 and PC2 scores plot (**d** adult **e** larvae). **f** PC1, PC2 and PC3 *C. elegans* and *P. pacificus* developmental stages. **g** PC1&2, PC1&3 and PC2&3 scores biplots for *C. elegans* and *P. pacificus* developmental stages. PC1, PC2 and PC3 loadings plots , where blue and red dashed lines indicate 1 and 2 standard deviations from the mean, respectively (**h** adults **i** larvae and **j** adults & larvae).

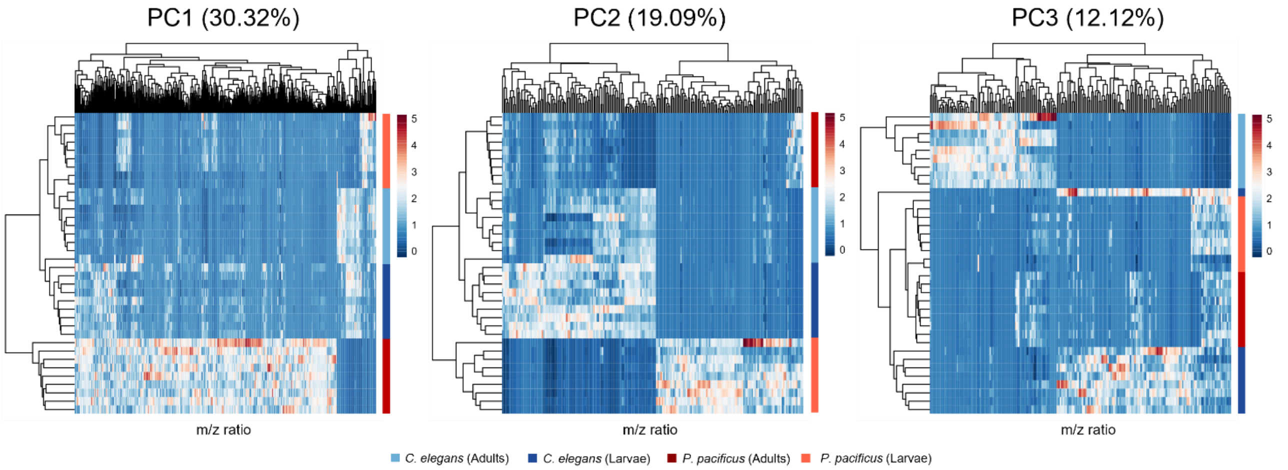

Fig. S7 | Comparing *C. elegans* and *P. pacificus* hierarchical clustering heatmaps. Hierarchical clustering heatmaps for subsets of data exhibiting loadings greater than one standard deviation from the mean using PC1-3 PCA analysis.

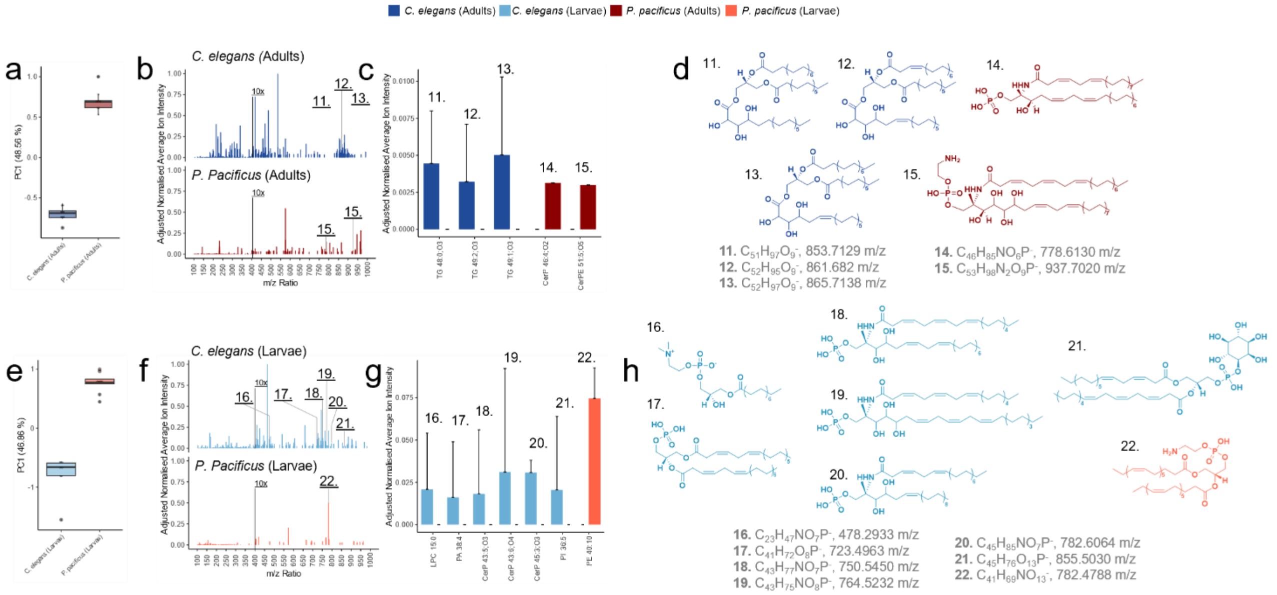

Fig. S8 | Species-specific surface chemical contrast divergence. PCA PC1 scores plot for *C. elegans* and *P. pacificus* (**a** adults and **e** larvae). Averaged surface secondary ion mass spectra exclusive to *C. elegans* and *P. pacificus* (**b** adults and **f** larvae) . Putative chemical assignments (**c** adults and **g** larvae) and structures (**d** adults and **h** larvae) on *C. elegans* and *P. pacificus* surfaces (P<0.001 by Student's t-test, n=9), present in LIPIDS MAPS (M-H, < 2ppm).

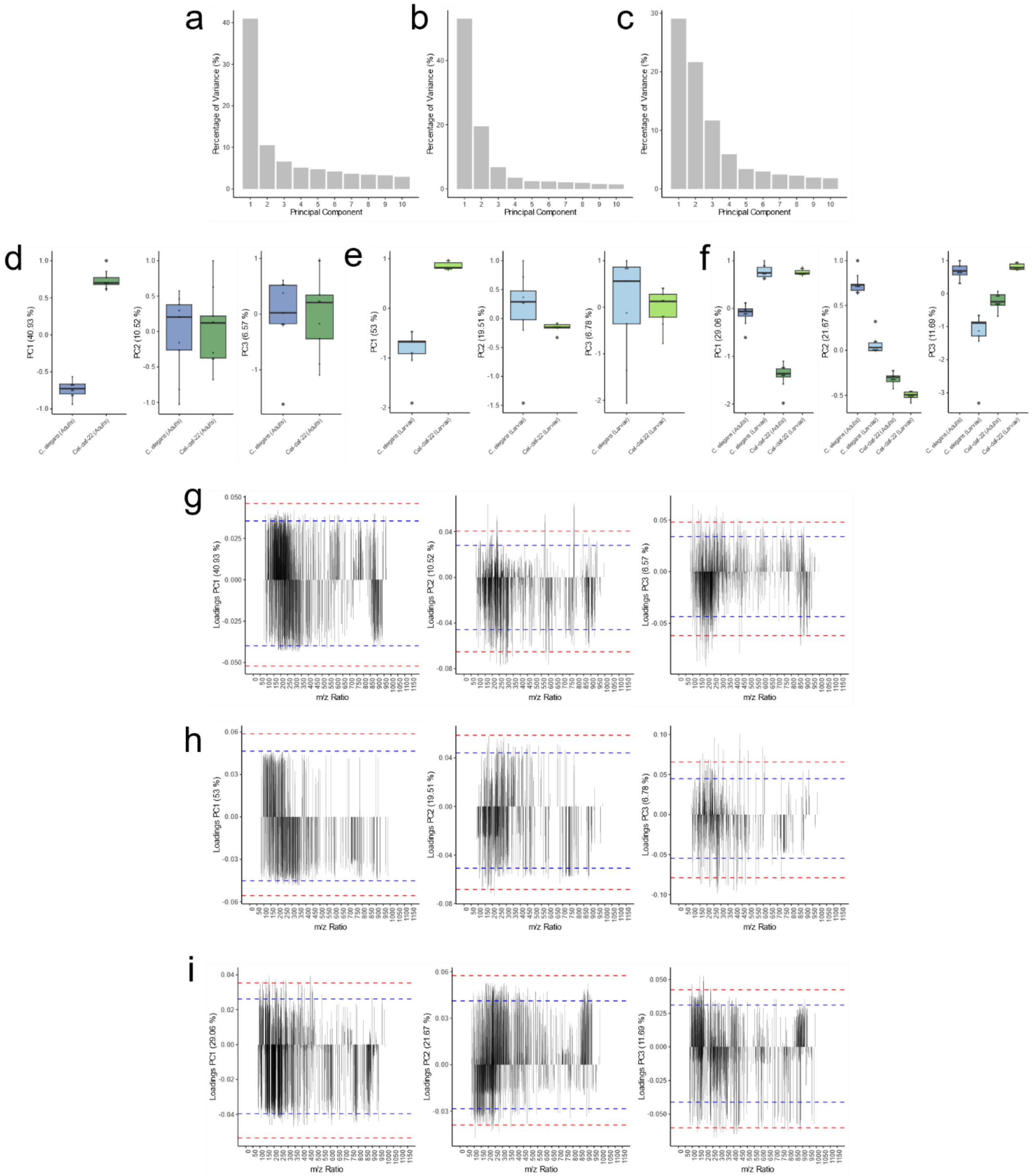

Fig. S9 | Multivariate analysis of *C. elegans* wildtype and *daf-22* for each developmental stage. a Percentage of variance on each principal component (**a** adults, **b** larvae and **c** adults & larvae). PC1, PC2 and PC3 scores plot (**d** adults and **e** larvae). **f** PC1, PC2 and PC3 wildtype and daf-22 developmental stages scores plot. PC1, PC2 and PC3 loadings, where blue and red dashed lines indicate 1 and 2 standard deviations from the mean, respectively (**g** adults, **h** larvae and **i** adults & larvae)

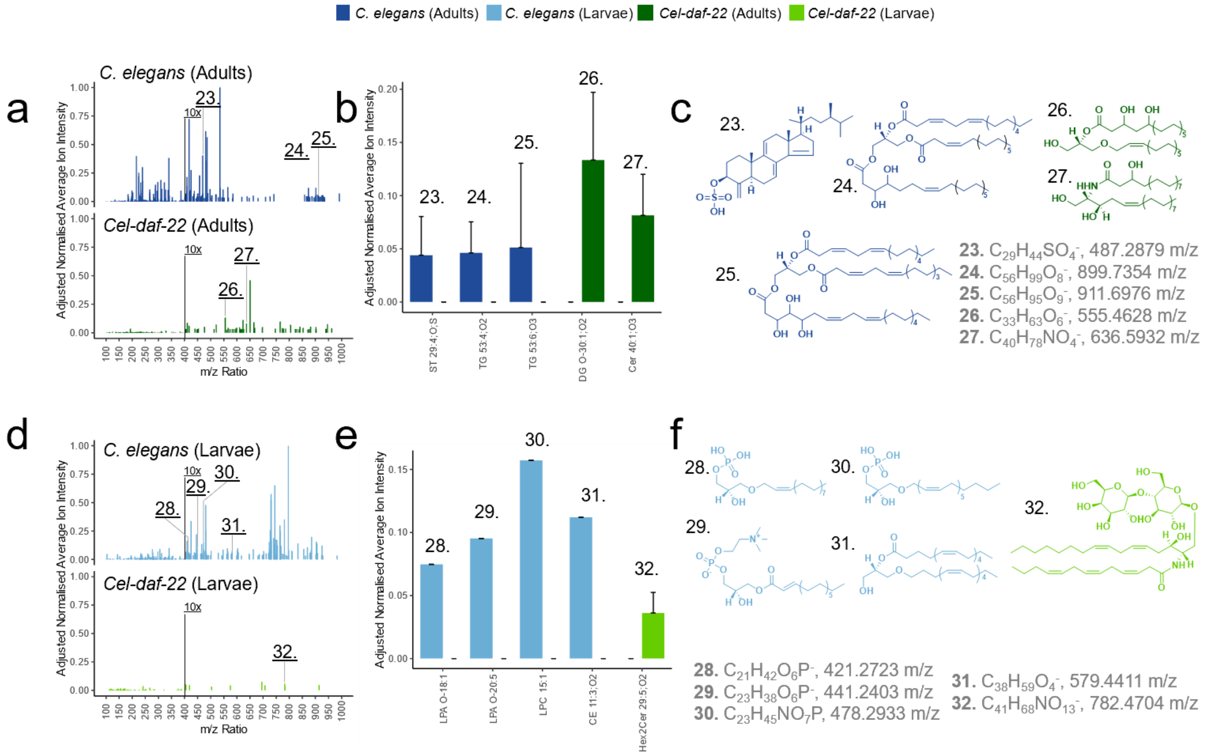

Fig. S10 | Putative *C. elegans* wild-type and *daf-22* mutant chemical assignments and structures Averaged surface secondary ion mass spectra exclusive to *C. elegans* and *Cel-daf-22 (***a** adults and **d** larvae). Putative chemical assignments (**b** adults and **e** larvae) and structures (**c** adults and **f** larvae) on *C. elegans* and *Cel-daf-22* surfaces (*P*<0.001 by Student's t-test, n=9), present in LIPIDS MAPS (M-H, < 2ppm).

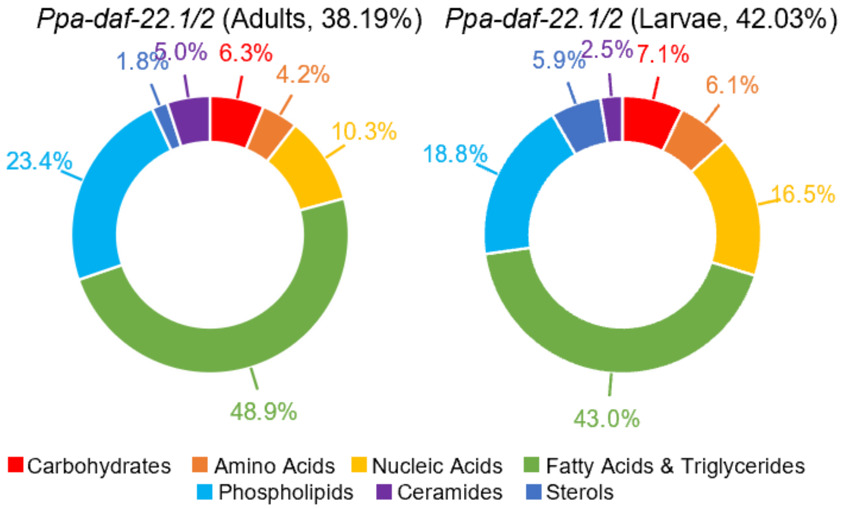

Fig. S11 | *P. pacificus* surface profile is dependent on *daf-22*. Distribution of molecular assignments determined using chemical filtration (**Table S1**), as a percentage of total ions surveyed for *Ppa-daf-22.1/2* adults and larvae surfaces.

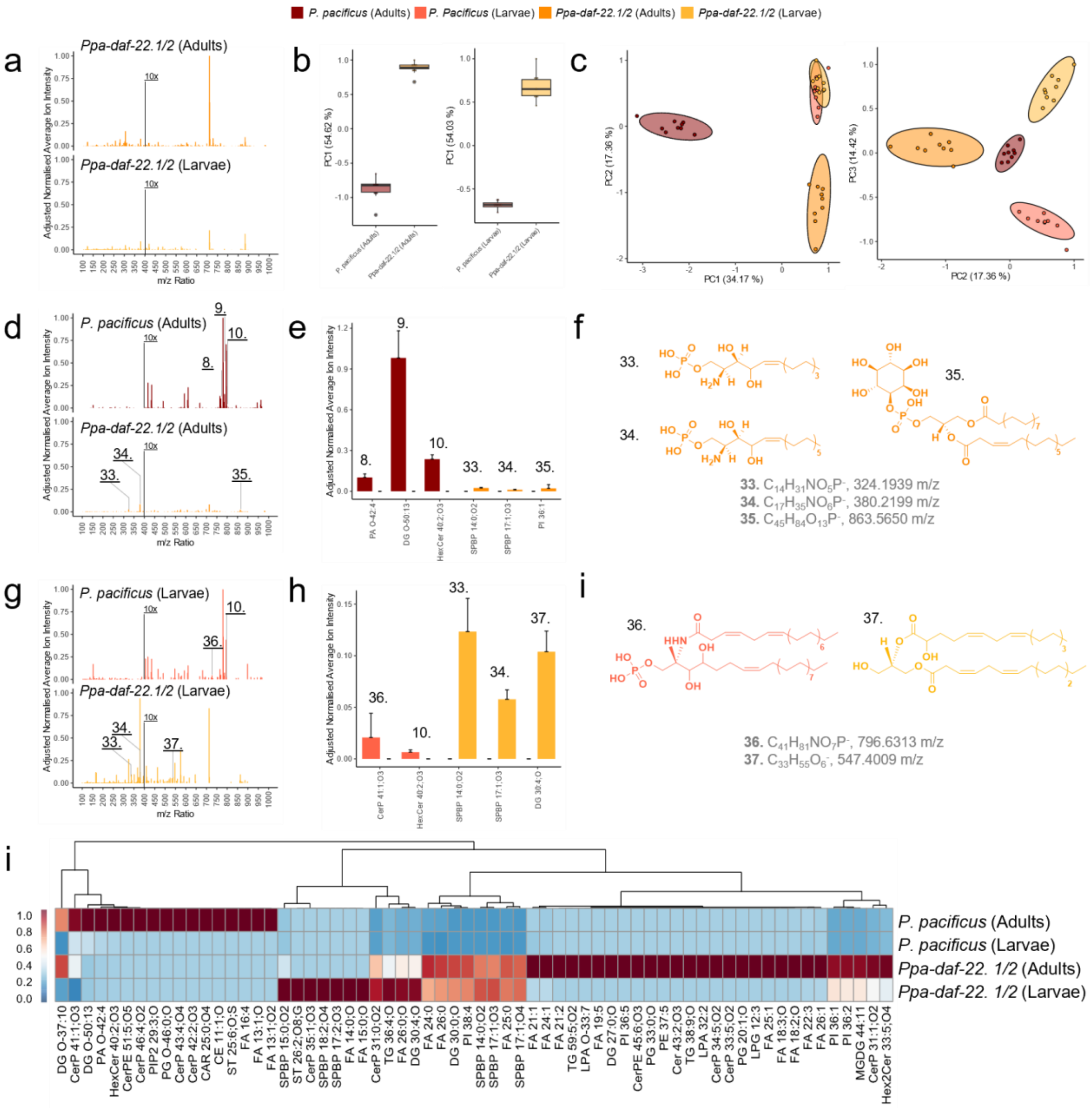

Fig. S12 | *P. pacificus* surface profile is dependent on *daf-22*. a Averaged *Ppa-daf-22.1/2* adults and larvae surface secondary ion mass spectra, normalised to maximum intensity across spectra, where intensity *m/z* >400 enhanced 10x for visibility. PCA **b** PC1 and **c** PC1 & PC2 and PC2 & PC3 scores plots for *P. pacificus* and *Ppa-daf-22.1/2* developmental nematode stages. Averaged surface secondary ion mass spectra exclusive to *P. pacificus* and *Ppa-daf-22.1/2* (**d** adults and **g** larvae). Putative chemical assignments (**e** adults and **h** larvae) and structures (**f** adults and **i** larvae) on *P. pacificus* and *Ppa-daf-22.1/2* surfaces (*P*<0.001 by Student's t-test, n=9), present in LIPIDS MAPS (M-H, < 2ppm). **j** Exclusive *P. pacificus* wildtype and *Ppa-daf-22.1/2* mutant chemistries, where chemometric clustering indicates potential shared regulation of exclusive chemicals and their relative intensity on nematode surfaces.

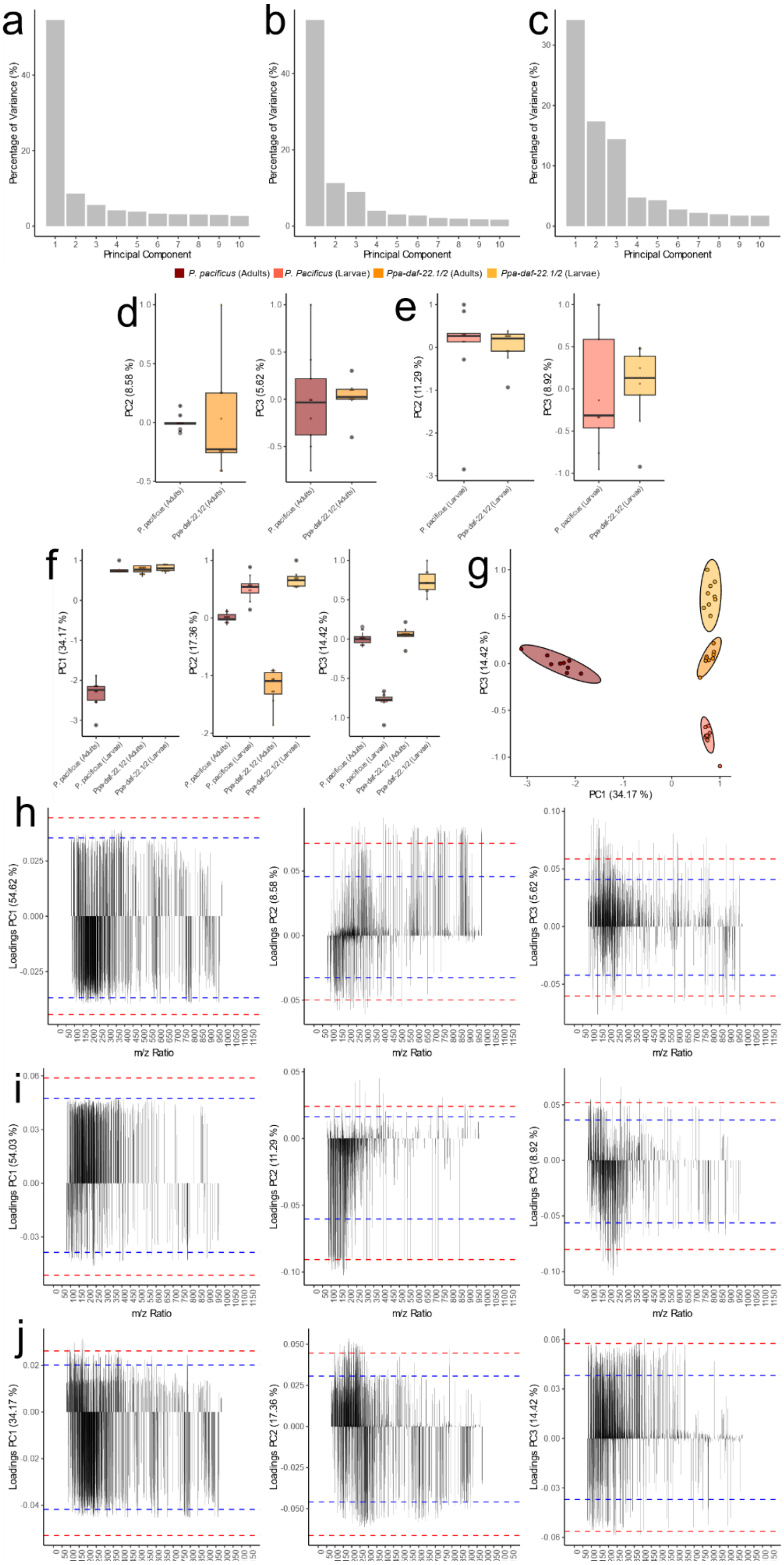

Fig. S13 | Multivariate analysis of *P. pacificus* wildtype and *daf-22* for each developmental stage. Percentage of variance on each principal component (**a** adults, **b** larvae and **c** adults & larvae). PC2 and PC3 scores plot (**d** adults and **e** larvae). **f** PC1, PC2 and PC3 wildtype and daf-22 developmental stages scores plot. **g** PC1&3 scores for *P. pacificus daf-22* developmental stages PC1, PC2 and PC3 loadings , where blue and red dashed lines indicate 1 and 2 standard deviations from the mean, respectively (**h** adults, **i** larvae and j adults & larvae)

Table S1 | Elemental composition ranges of key biomolecules. A comparative overview of the minimum and maximum counts of key elements in primary biological molecules.

CATEGORY	GENERAL FORMULA	H	C	N	O	P	S
**Carbohydrates**	$Cn(H_2O)n-1$	2-12	1-7	0	1-6	0	0
**Proteins (Amino Acids)**	-(Amino Acid)n	2-17	1-8	1	1-3	0	0-1
**Nucleic Acids**	-(Nucleotide)n-1	2-9	1-8	1-3	1-6	1	0
**Fatty Acids & Triglycerides**	$CH_2(CH_2)nCOO-$ / $C_{55}H_{97}O_5$	4-90	2-50	0	1-4	0	0
**Phospholipids**	$R_1COOR_2R_3PO_4R_4$	8-80	4-40	1	4-8	1-2	0
**Ceramides**	$C_{34}H_{65}NO_2$	5-60	9-30	1	1	0	0
**Sterols (e.g., Cholesterol)**	$C_{27}H_{45}O-$	9-44	10-25	0	1	0	0

Supporting Spreadsheet S1 | Isobaric assignments for nematode surface lipid composition. This spreadsheet provides a comprehensive list of isobaric assignments for each exact mass detected on nematode surfaces, matched with entries in the LIPID MAPS database (M-H, <2 ppm). For each detected mass, potential lipid candidates are listed along with their exact masses, delta (mass error), and putative lipid assignments. This data offers detailed insights into possible molecular identities, facilitating interpretation of surface lipid profiles in *Caenorhabditis elegans* and *Pristionchus pacificus* while acknowledging isobaric limitations.

Supporting Video S1 | *P. pacificus predation.* Adult *P. pacificus* wildtype predating on *C. elegans* wildtype larvae.

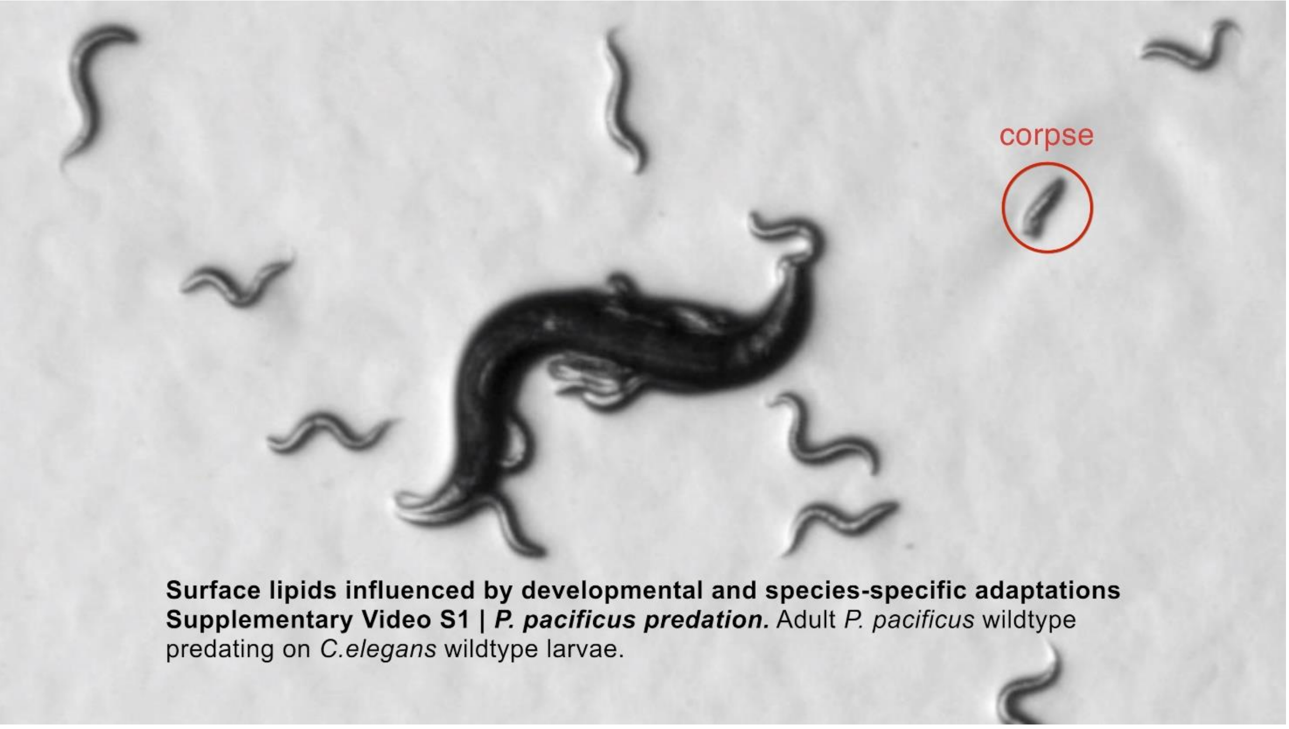

Supplement: Supplementary file 1 — ja4c12519_si_001.pdf [file ja4c12519_si_001.pdf]
